# Machine Learning Techniques Applied to Multiband Spectrum Sensing in Cognitive Radios

**DOI:** 10.3390/s19214715

**Published:** 2019-10-30

**Authors:** Yanqueleth Molina-Tenorio, Alfonso Prieto-Guerrero, Rafael Aguilar-Gonzalez, Silvia Ruiz-Boqué

**Affiliations:** 1 Master of Sciences and Information Technologies, Metropolitan Autonomous University Iztapalapa, Mexico City 09360, Mexico; yanqueleth@xanum.uam.mx; 2 Electrical Engineering Department, Metropolitan Autonomous University Iztapalapa, Mexico City 09360, Mexico; r.aguilar@xanum.uam.mx; 3 Department of Signal and Theory Communications, Universitat Politècnica de Catalunya, 08860 Barcelona, Spain; silvia.ruiz@upc.edu

**Keywords:** cognitive radios, multiband spectrum sensing, machine learning, neural networks

## Abstract

In this work, three specific machine learning techniques (neural networks, expectation maximization and *k*-means) are applied to a multiband spectrum sensing technique for cognitive radios. All of them have been used as a classifier using the approximation coefficients from a Multiresolution Analysis in order to detect presence of one or multiple primary users in a wideband spectrum. Methods were tested on simulated and real signals showing a good performance. The results presented of these three methods are effective options for detecting primary user transmission on the multiband spectrum. These methodologies work for 99% of cases under simulated signals of SNR higher than 0 dB and are feasible in the case of real signals.

## 1. Introduction

Spectrum scarcity has been an issue in past years, due to the increasing demand for wireless communications services. Several instances have demonstrated that a solution is highly necessary in order to provide spectrum spaces for future technologies. In this regard, among engineering and regulatory proposed solutions, cognitive radio (CR) has been an outstanding option. CR is based on software-defined radio (SDR) and dynamic spectrum access (DSA) technologies, both allowing to improve drastically the spectrum occupancy [1,2]. Users that consider the utilization of CR are known as secondary users (SU). They do not have assigned a spectrum space; however they have the capacity to use available spectrum spaces adapting to the environment, which provides an affordable communication service. This process should be done without provoking an interference to the licensed users, called also primary users (PU) [3].

In order to accomplish its promises, CR has to perform mainly four tasks such as *spectrum sensing, spectrum sharing, spectrum decision, and spectrum mobility* [4]. The primary task is spectrum sensing (SS), this activity is fundamental to detect PU presence and to determine if a spectrum space is occupied or empty. SS has been a widely studied topic, present in the literature with many different techniques, being most of them based on single band SS [5]. However, future communications services require high data rates, which can be reached only by considering non-contiguous spectrum bands. The last means that single band SS algorithms should be improved to include multiple bands, this paradigm is called multiband spectrum sensing (MSS).

According to the literature, a branch of MSS techniques corresponds to wide-band spectrum sensing (WBSS) [6]. At the same time, WBSS is divided into sub-categories, for example: wavelet-based spectrum sensing, compressive sensing, and angle-based spectrum sensing among others. Several important contributions related to MSS are mentioned in [7,8,9]. All these methods and techniques are considered in order to improve the performance of SS and CR. 

Authors of this paper presented a previous work describing how to detect efficiently PU presence by considering new alternative methods [10], based on hybrid linear and non-linear techniques. In this contribution, a couple of MSS algorithms based on multiresolution analysis (MRA) and the Higuchi fractal dimension (HFD) were proposed and discussed. However, there are several basic issues which still represent a challenge, for example, the threshold setting for distinguishing PU presence or absence [11] and the new paradigm to convert CR in an really smart entity [12]. In [10], the detection threshold of PUs was manually placed, derived from the results obtained with simulated signals. Nevertheless, considering the high variability of the spectrum occupancy, this option was not optimal. Therefore, in this paper, a new proposal is analyzed, reconsidering the way in which the CR should act to automatically place the optimal threshold while dynamically adapting it to follow this spectrum occupancy. To do so three different machine learning (ML) algorithms are proposed, implemented and compared, showing that they are useful and efficient techniques to detect occupied or empty bandwidth spaces in a multiband spectrum sensing scenario.

Previous works have included ML techniques in their CR proposals. In [13], it is mentioned an overview of the ML techniques that have been used for different CR tasks. SS and MAC protocols have used reinforcement learning (RL) because is the optimal solution for Markov decision process and game theory-based learning. For signal classification and feature detection non-parametric learning, Dirichlet Process mixture model has been used, given that does not require prior knowledge about the number of mixture components. Artificial neural networks (NN) do not require prior knowledge of the distribution of the observed process. For the case of support vector machines (SVM) algorithm, which has better performance for small training examples compared to artificial NN, it requires prior knowledge of the distribution of the observed process and requires data labeling. For power allocation and rate adaptation, it is considered the theory-based learning strategy. Finally, for the reconfiguration of system parameters, the threshold learning is used, which is suitable for controlling specific parameters under uncertainty conditions with the restriction of training data.

Interesting works for CR and ML techniques are available in the literature. For example, in [14] a cooperative spectrum sensing (CSS) scheme based on ML techniques applied to an energy vector is proposed, where each component is an energy level estimated at each CR device. Results show how CSS techniques, considering ML, are capable of implicitly learning from the surrounding environment. In [15], an ML-based multiband spectrum sensing policy has been proposed using the greedy method to track the occupancy statistics of PUs and to estimate the detection performance of the SUs. The policy considering the greedy method can select those sub-bands which provide spectrum opportunities with high throughput for the secondary network. In [16], a technique for sensing the primary radio signal in a cognitive environment using a learning algorithm based on artificial NN is analyzed. In [17], a channel state predictor is implemented for multi-SU in a CR using NN. The Dirichlet process has been used as a framework for non-parametric Bayesian learning in CRs in [18].

A contribution related to SS and ML appears in [19], where a scheme based on the algorithm *K*-nearest neighbors is proposed. The method includes the training and classification phase, and each user takes a decision that is processed in a fusion center. The results show advantages in the detection of PU compared to traditional methods. In [20] the spectrum occupancy is analyzed by several supervised and unsupervised ML techniques, concluding that SVM is the best classification technique. Also, the SVM technique has shown good results in the allocation of resources such as power and channel, in cognitive radio networks [21].

This work is organized as follows: in Section 2, the theoretical bases of the implemented ML techniques are briefly presented. After, in Section 3, a summary of the methodology of our previous work is described. Besides, in this same section, the new methodology considering the ML techniques is explained. Section 4 shows the simulation environment and results. Finally, Section 5 mentions the conclusion and a brief discussion.

## 2. Theoretical Bases of the Implemented ML Techniques

Machine learning provides computers with the ability to learn without being explicitly programmed. ML methods are very effective when the data set is large, diverse and fast-changing. These algorithms give a deep and predictive analysis of data [22] and can be classified into two big groups: supervised (clustering techniques) and unsupervised learning (classification and regression techniques). This section describes briefly the background of the techniques associated with machine learning that will be implemented in the analysis.

### 2.1. Neural Networks

**Figure 1:**
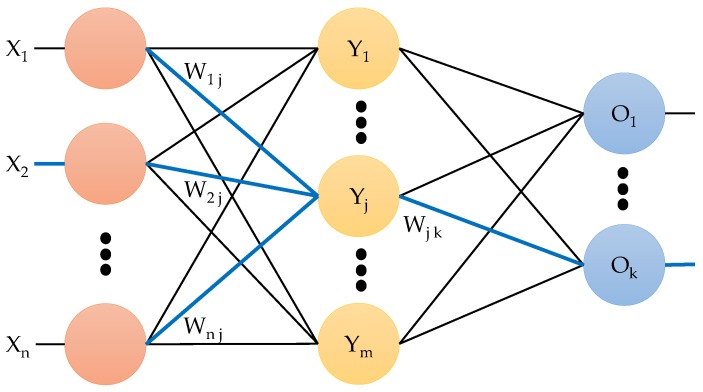
Neural network concept.

Artificial NN are computational models inspired by the central nervous system, specifically the brain. NN have the capacity to perform pattern recognition. NN usually are presented as a system of interconnected “neurons” that can compute values from inputs by feeding information through the network [23]. The NN are supervised algorithms that require training with labeled data. These networks are based on empirical risk minimization and they require prior knowledge of the observed process distribution. Figure 1 shows a NN consisting of an input layer, one or more hidden layers and an output layer. A NN is used for both classification and prediction considering a back-propagation algorithm by weight adjustment of each edge of layers.

### 2.2. K-Means

The *K*-means algorithm allows the classification of a data set. This algorithm requires prior knowledge of the number *K* of groups to be classified. *K*-means defines *K* number of centroids, finds a partition that minimizes the squared error between the empirical mean of the centroid of a cluster and the points in the group. Iteratively recalculates the centroid of each cluster until finding a partition that reaches the convergence [24]. *K*-means usually converges to a local minimum, but in [25], it has been shown that when the clusters are well separated, it could skip the local minimum converging to the global one.

### 2.3. Expectation-Maximization

The Expectation-Maximization (EM) algorithm consists of two major steps: an expectation step, followed by a maximization step. The expectation is with respect to the unknown underlying variables, using the current estimate of the parameters and conditioned upon the observations. The maximization step provides a new estimation of the parameters. These two steps are iterated until convergence. The EM algorithm was discovered and employed independently by several different researchers. However, the authors of [26] brought their ideas together, proved convergence, and coined the term “EM algorithm”. In this work, EM is used to delimit 2 events and can be described as follows.

Given $x_{1},\ldots ,x_{n}$ number of observations belonging to K types of groups. K Gaussians with $\left(\mu ,\sigma^{2}\right)$ are created randomly. In this work only two events (K=2) are considered, resulting in $G1(\mu_{1},\sigma_{1}^{2})$ and $G2(\mu_{2},\sigma_{2}^{2})$. For each point $x_{i}$, $P(b|x_{i})$ is calculated. Where b={A,B} are the considered events:

(1) $$P(B|x_{i})=\frac{P(x_{i}|B)\;P(B)}{P(x_{i}|B)\;P(B)+P(x_{i}|A)\;P(A)}$$

(2) $$P(x_{i}|B)=\frac{1}{\sqrt{2\pi }\sigma_{B}^{2}}\exp(-\frac{{\left(x_{i}-\mu_{B}\right)}^{2}}{2\;\sigma_{B}^{2}})$$

Recalculating $\left(\mu_{1,2},\sigma_{1,2}^{2}\right)$ for A and B:

(3) $$\mu_{1}=\frac{b_{1}x_{1}+\ldots +b_{n}x_{n}}{b_{1}+\ldots +b_{n}}$$

(4) $$\sigma_{1}^{2}=\frac{b_{1}{\left(x_{1}-\mu_{b}\right)}^{2}+\ldots +b_{n}{\left(x_{n}-\mu_{n}\right)}^{2}}{b_{1}+\ldots +b_{n}}$$

Analogously with $\left(\mu_{2},\sigma_{2}^{2}\right)$.

## 3. ML-Based Methodology

**Figure 2:**
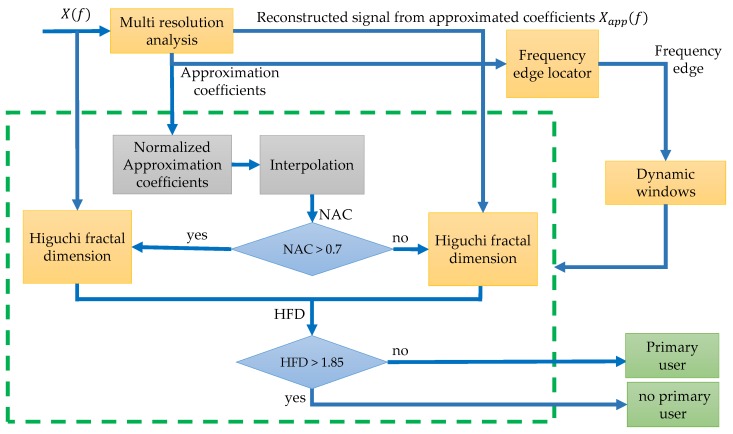
The second methodology described by a flow diagram, developed in [10].

First, it is presented a short summary of the technique developed by the authors in [10]. Basically, this original methodology considers the multiresolution analysis (a wavelet-based dyadic filter bank) and the Higuchi fractal dimension to detect transmissions of PUs. The flowchart of the implemented methodology of this previous work is described in Figure 2 where X(f) represents the power spectral density in a wide frequency range or the received multiband spectrum by a SU.

This original methodology is described by the following steps:

Step 1.The received multiband spectrum by a SU, X(f), is decomposed via the MRA at defined level 3 with a Haar wavelet, giving the respective approximation and details coefficients.Step 2.From the obtained approximation coefficients at level 4, the spectrum is reconstructed eliminating in this way the broadband noise and only keeping the *tendency* (or smooth shape $X_{app}\left(f\right)$) of the multiband spectrum X(f).Step 3.The frequency edges locator is constructed by detecting the values changes of the same approximation coefficients passing through the threshold of 0.7, i.e., the approximation coefficients going from down to up of the 0.7 or vice versa. These changes will be the frequency edges necessary to construct the dynamic windows for further analysis.Step 4.The same approximation coefficients obtained in Step 1 are normalized e interpolated (to have the same samples that the dynamic windows). With these normalized and interpolated approximation coefficients (NAC), each conformed window is then processed to detect noise or a possible PU transmission. If the NAC are, on average, under a defined threshold of 0.7 then the values of the analyzed windows is probably a PU transmission. If NAC is greater than 0.7 is practically sure that the transmission corresponds to noise.Step 5.If NAC is greater than 0.7 then the Higuchi fractal dimension is applied directly on the analyzed section of the multiband spectrum X(f). In the other case, the HFD is applied to the reconstructed signal from Step 2 (i.e., $X_{app}\left(f\right)$).Step 6.For each window, if the calculated HFD is lower than 1.85 (decision threshold) a PU transmission is detected. In another case, no PU is detected (only noise).

The inclusion of ML techniques in the original methodology described above has the main goal to improve the detection process of PUs. Basically, the two first steps and the last one of the previous work remain the same, however, in this new approach the NAC are processed by ML techniques improving the detection process before applying the HFD, modifying the original methodology in Steps 3 and 4 (marked by a dashed red line). The threshold of 0.7 is modified with these ML techniques and instead of being fixed to a constant value, is adapted dynamically enhancing the frequency edges. 

**Figure 3:**
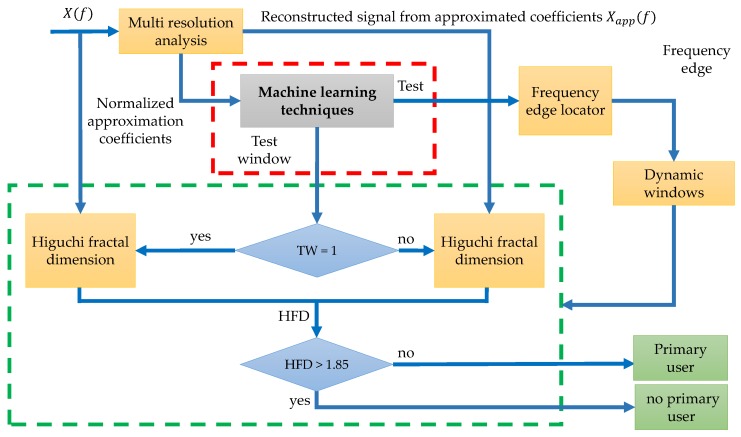
Improving of the original methodology in [10] using ML techniques.

Figure 3 shows the introduction of this new block, and the red dashed line points out the place for ML techniques. All techniques, described in Section 2, are implemented in this block. As a result, the frequency edges are improved and a Test window (TW) is used to determine the presence or absence of PUs instead of NAC directly (Step 5 also marked by a dashed red line).

Next, the implementation of each ML techniques is described starting with the training process for detection of PUs, and describing later how each technique is introduced in the original methodology. 

### 3.1. Neural Network with Manual Threshold Setting

**Figure 4:**
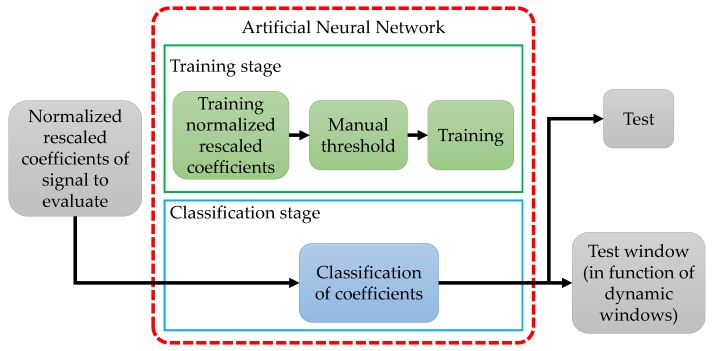
The general scheme for the NN-based technique with a manual threshold setting.

In this work, an NN is used as a classifier. This classifier uses the normalized approximation coefficients to determine the presence of PU. To make a better detection, the approximation coefficients for the signals with PU transmissions are rescaled in the interval of [0, 1], where 0 represents the coefficient with more power and 1 represents the coefficient with less power. Figure 4 shows the implemented scheme for this ML technique.

**Figure 5:**
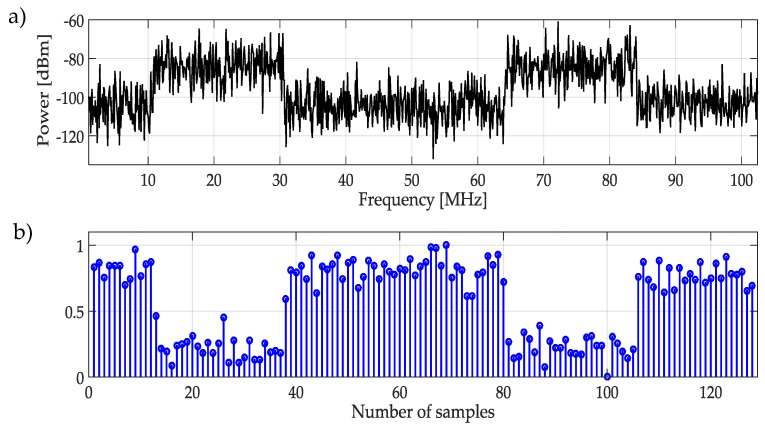
(**a**) Random signal used for the NN training, (**b**) Normalized and rescaled approximation coefficients which are used to train the NN.

First, the NN is trained based on a single frame from the whole wideband spectrum, i.e., X(f) and its MRA. The considered frame for training is chosen randomly but *must* contain one or more PU transmission. Figure 5a shows an example of the signal used for the NN training, while Figure 5b represents the normalized and rescaled approximation coefficients, obtained from the MRA that will be used to train the NN. This step provides to the NN, information about frequency band appearance with and without PUs activities. To separate both classes (PUs or SUs), it is necessary to set an initial threshold before NN training. A *manual threshold* was initially proposed, but this means that the threshold setting will depend on the criteria of an external user, making the process extremely unpredictable. However, for a signal with a large difference among coefficients (low noise), the threshold setting is not a big deal.

**Figure 6:**
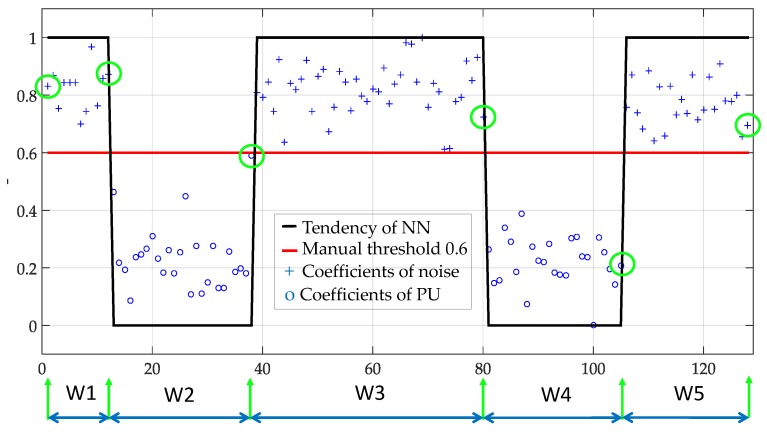
The result of training the NN with a manual threshold set at 0.6 over an entire frame. The normalized and rescaled approximation coefficients have been classified. Also, the borders of dynamic windows are determined.

In Figure 6, the user set the threshold at 0.6, and the NN was trained. In this figure, according to this *manual threshold*, the approximation coefficients were classified and represented with 1 for noise and 0 for PUs, identifying correctly the PUs presence. Thus, the trend of coefficients is clear and helps, besides to separate both classes, to determine the frequency edges of a PU transmission. This occurs when a change is present from a coefficient associated to noise and the immediately next is a coefficient associated to a PU (or vice versa), as can be shown by the green circles in Figure 6. These values (green circles) will permit to construct the dynamic windows (marked as W1,…,W5 in this case) that will be further processed individually to determine if the signal segment corresponds or not to a PU.

With the NN trained, now it is possible to apply this NN to other frames and obtain the frequency edges from the normalized and rescaled approximation coefficients. These frequency edges permit to build the dynamic windows. According to Figure 3, prior to decide if applying the HFD to X(f) or $X_{app}(f)$, it is necessary to do the classification of the coefficients, establishing the “test signal” as the complete output resulting by the trained NN and the “test window” as the specific segments of the test signal associated to dynamic-sized windows. Steps 3 to 5 of the original methodology have been modified as:

Step 3.A training frame is selected: the approximation coefficients are normalized and rescaled. These coefficients are used to train the NN with an initial threshold set by the user. After NN training, the normalized and rescaled approximation coefficients from another random analysis frames are obtained and evaluated by the trained NN, resulting in the test which contains the classification of coefficients (0 for a possible PU transmission and 1 for noise).Step 4.The frequency edges and dynamic windows are determined from the output of the NN. The edges are the result of the change from one state to another (from one to zero and vice versa).Step 5.If the test in each dynamic window (spectrum signal segment) corresponds to 1, it is practically sure that the transmission corresponds to noise, that means the HFD will be applied to X(f). On the contrary case, the analyzed windows are highly probably a PU transmission and the HFD will be applied to $X_{app}\left(f\right)$.

**Figure 7:**
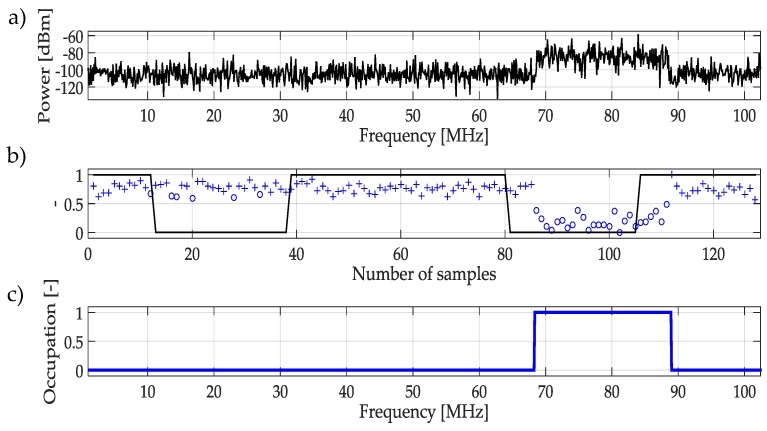
(**a**) Real random multiband signal. (**b**) Classification of coefficients, the result of using the trained NN mentioned in Figure 6. (**c**) Result of applying the methodology using NN as a classifier.

The result of this whole process is shown in Figure 7. First, in Figure 7a a real random multiband signal is presented, this signal is just an example to demonstrate the functionality of the methodology. Figure 7b shows the classification of the approximation coefficients using the NN. Here, it is possible to appreciate the difference between coefficients associated with PU and noise. Finally, in Figure 7c the result of the determination of the availability of a bandwidth space is indicated. In this figure, it is clear how the trained NN classifies the spectrum signal is occupied or empty.

### 3.2. Neural Network with an EM Threshold Setting

**Figure 8:**
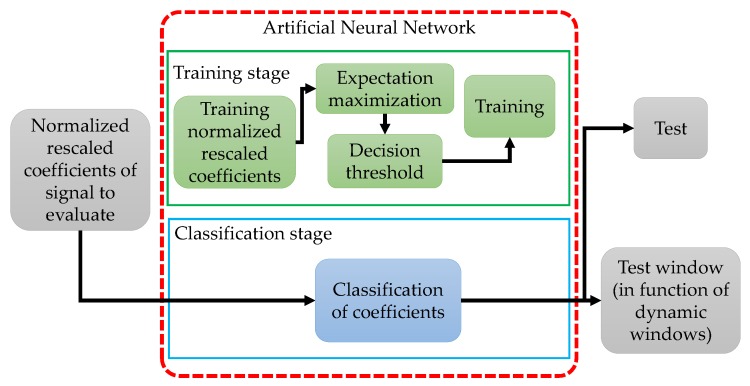
The general scheme for the NN-based technique with a threshold setting using EM.

The NN training threshold that was used in Section 3.1 depends on the criteria of the user. To improve it, an Expectation Maximization (EM) algorithm is included to select automatically and optimally the threshold. Figure 8 shows the new block diagram including EM.

**Figure 9:**
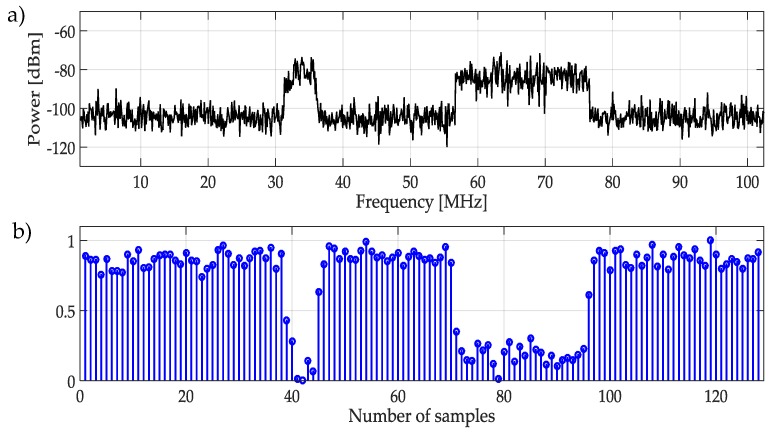
(**a**) Multiband spectrum generated randomly. (**b**) Approximated, normalized and rescaled approximation coefficients which are used to train the NN.

Figure 9 shows the normalized and rescaled approximation coefficients of a generated random multiband spectrum used to train the NN. When applying EM to these coefficients, two specific limits are obtained. The threshold is then selected randomly between these limits (a detailed explanation of this decision is explained in Section 4.1).

**Figure 10:**
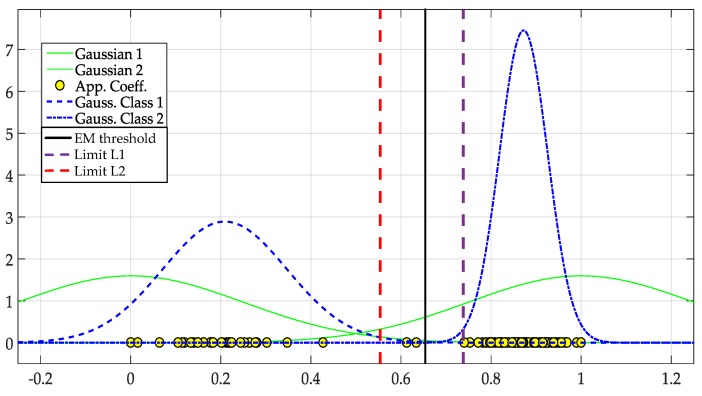
The power of the approximation coefficients in axis X and the result to classify them in two classes.

In Figure 10 the application of the EM algorithm is exemplified. Here, the first two classes are randomly chosen with Gaussian distribution (marked in green), the normalized and rescaled approximation coefficients are plotted on the X axis (marked with yellow bullets). The result of applying EM to these coefficients are the two Gaussians in blue, where it is clearly seen that they correspond to two groups that can be separated. The limits L1 and L2 are set at $L1=\mu_{1}+3\sigma_{1}$ and $L2=\mu_{2}+3\sigma_{2}$ when L1<L2 since the probability of occurrence is 0.001 for each class and where (0.211, 0.1143) and (0.872, 0.0446) are the mean and standard deviation (STD) of Gaussians 1 and 2, respectively. Finally, the threshold is chosen randomly between L1 and L2. The uncertainty between what the optimal value would be is clarified in Section 4. When L1>L2 means that the SNR is close to 0 dB. In this case, $L1=\mu_{1}+n\sigma_{1}$ and $L2=\mu_{2}+n\sigma_{2}$ are adjusted with n=2,1,0 until L1<L2 where the probability of occurrence for each n would be 0.022,0.158,0.499.

**Figure 11:**
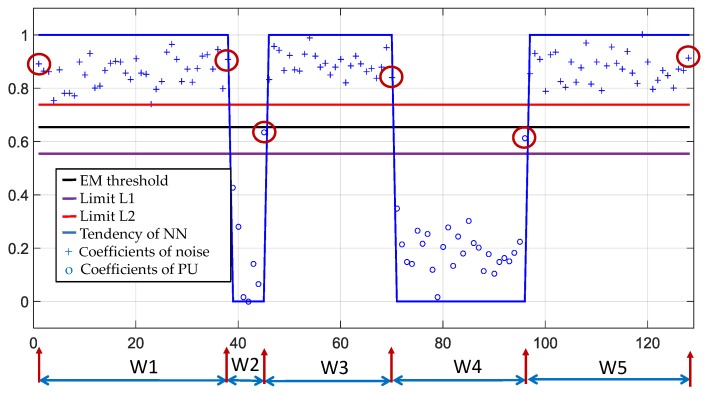
The result of training the NN with the EM-based threshold set to 0.654. Determination of frequency edges and construction of the dynamic windows.

In the case of the analyzed example (Figure 9), the threshold is set to 0.654 (Figure 10). Applying this threshold to a training signal allows the classification of coefficients and the determination of frequency edges that facilitate the building of the respective dynamic windows as it is shown in Figure 11. This improvement only modifies Step 3:

Step 3.A training frame is selected: the approximation coefficients are normalized and rescaled. These coefficients are used to train the NN where coefficients will be classified and two limits (L1 and L2) estimated by the EM algorithm. Then, a random value between L1 and L2 is selected as threshold.
After NN training, the normalized and rescaled approximation coefficients from another random analysis frames are obtained and evaluated by the trained NN, resulting in the test which contains the classification of coefficients (0 for a PU transmission and 1 for noise).

**Figure 12:**
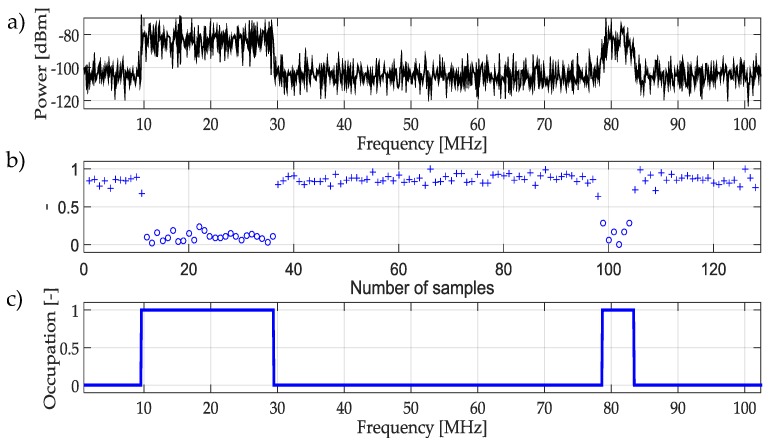
(**a**) Multiband spectrum generated randomly. (**b**) Classification of the approximation coefficients, the result of using the trained NN mentioned in Figure 11. (**c**) Result of applying the complete methodology using a NN as a classifier.

The result of applying this technique is shown in Figure 12. Here, a multiband random signal is used to determine its states of occupancy based on the mentioned trained NN clearly detecting the presence of PUs.

### 3.3. Methodology Using a K-Means Classifier

**Figure 13:**
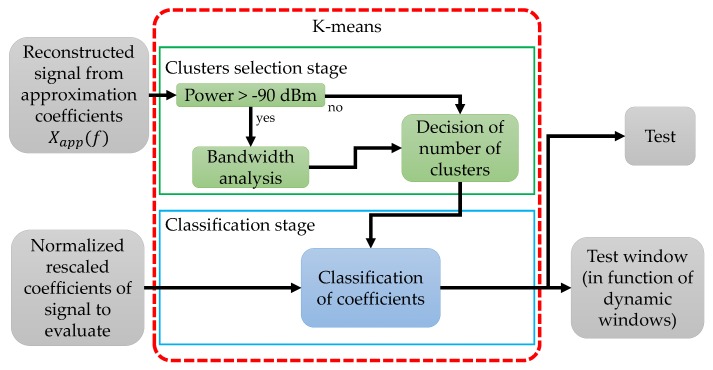
The general scheme for the approximation coefficients classification based on the *K*-means technique.

Another unsupervised method used in this work as a classifier of the approximation coefficients from a random multiband spectrum is the *K*-means technique. In order to determine the multiband availability of the spectrum signal, the approximation coefficients, normalized and rescaled, are first classified with *K*-means. The flowchart of this process is shown in Figure 13.

In this technique, the objective is to classify the approximation coefficients into 2 groups, one inferring the PU transmission and one corresponding to the noise, when the multiband spectrum contains PUs transmissions. On the other hand, they must be classified in a single group when the same radio-electric space contains only noise. However, one of the disadvantages found in *K*-means is that it is necessary to indicate the number of clusters that are expected, which introduces a difficulty since the frames that are evaluated by the algorithm are random. Then, to indicate how many clusters the *K*-means algorithm needs, it is necessary a prior analysis of the behavior of $X_{app}(f)$. As it is shown in Figure 13, the possible presence of a PU in the signal $X_{app}(f)$ is first evaluated setting a simple energy detector at −90 dBm. If there is no detection, the number of clusters is set to 1. On the other hand, if some transmission exceeds the threshold, we have three possible cases which are solved with a small analysis of bandwidth:

Case 1: impulsive noise in the signal. Owing to the small bandwidth of this anomaly, i.e., less than 7 samples * 0.1 [MHz], the number of clusters chosen is still 1.
Case 2: possible PU transmissions in the signal. With the understanding that a transmission has at least 7 samples * 0.1 [MHz] then the number of clusters will be 2.
Case 3: impulsive noise and possible PU transmissions coexisting in the frame. In this case, when locating that there is a possible transmission with at least 7 samples * 0.1 [MHz], that means the chosen number of clusters will be 2.

The methodology is modified in Steps 3 and 4, regarding the proposed steps in Section 3.1 as follows:

Step 3.A priori analysis directly on the approximation coefficients to select the number of clusters is done: If the reconstructed signal ($X_{app}(f)$) does not exceed −90 dBm, the cluster number will be 1 (only noise). Else, the bandwidth of the signals that exceeded this threshold is studied (3 cases mentioned before). To confirm that it is not impulsive noise (2 clusters).
Then, the normalized and rescaled approximation coefficients from another random analysis frames are obtained and evaluated by the *K*-means algorithm, resulting in the test which contains the classification of coefficients (0 for a PU transmission and 1 for noise).Step 4.The frequency edges and dynamic windows are determined from the output of the classifier. The edges are the result of the change from one state to another (from one to zero and vice versa).

**Figure 14:**
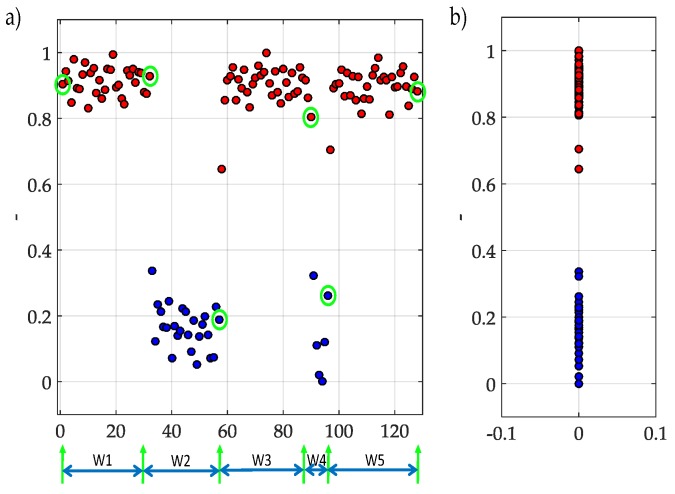
(**a**) Classification of coefficients with *K*-means and their respective frequency edges. (**b**) Power of coefficients in Y-axis the result of use *K*-means as a classifier.

The result of applying *K*-means to coefficients is shown in Figure 14. In Figure 14a we can observe how dynamic windows and the frequency edges are chosen. In Figure 14a, the clustering is given in two groups where the presence of PUs exists. In Figure 14b, it is shown the power of coefficients in Y-axis, where 1 corresponds to low power (bullets in red color) and 0 to a higher power (bullets in color blue). *K*-means classifies correctly the given the approximation coefficients, normalized and rescaled, of a random multiband spectrum.

**Figure 15:**
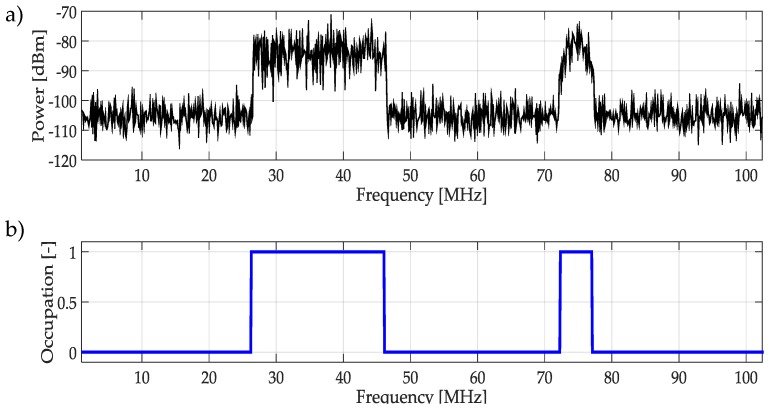
(**a**) The random multiband spectrum which corresponds to coefficients plotted in Figure 14. (**b**) The result of applying the complete methodology.

The result of applying *K*-means to the multiband spectrum monitoring technique using the technique as a classifier is shown in Figure 15, where Figure 15a corresponds to a randomly generated multiband spectrum, while Figure 15b shows the occupation along the frequency, result of applying the complete methodology.

## 4. Results 

In this section, the simulation environment to analyze and compare the pros and cons in the use of the three ML techniques is completely described. Performance is also checked using signals captured from a real environment. 

### 4.1. Simulated Signals

**Figure 16:**
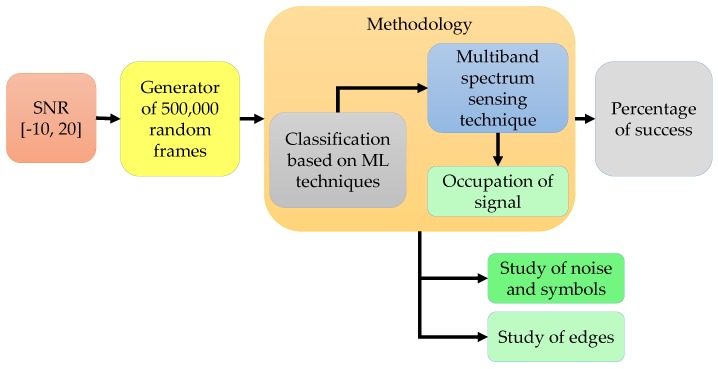
Scheme of the global simulation.

The methodology described in Section 3 with the ML techniques, was applied to a global simulation of 500,000 frames for SNR values in the interval [−10, 20] dB, as it is shown in Figure 16. In each frame, the number, type (OFDM or NRZ associated with CDMA) and position (frequency edges) of simulated symbols were generated randomly. Each frame consists of 1024 samples spaced by 0.1 MHz (i.e., an entire band consist of 102.4 MHz). Simulation parameters are summarized in Table 1. The analyzed blocks correspond to studies of noise, symbols, and frequency edges. These studies serve to analyze the behavior of symbols, noise and the accuracy of detected frequency edges.

**Figure 17:**
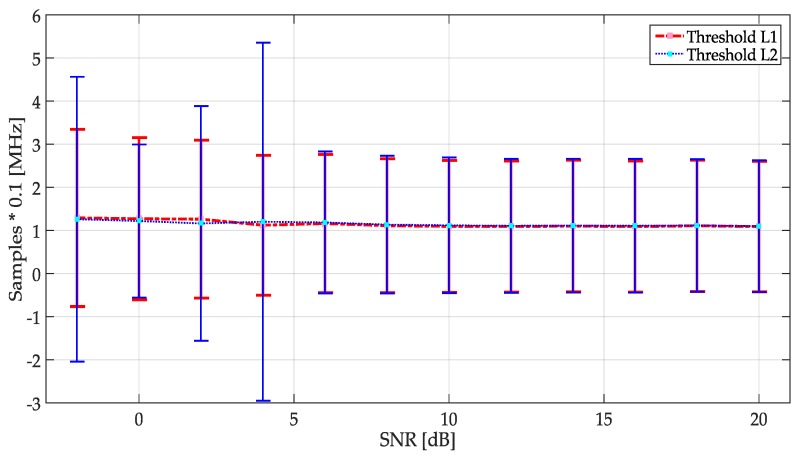
The mean and STD of the frequency edges location estimation using the threshold as *L*1 and *L*2 considered as the maximum and minimum values, respectively, for the NN training.

From Section 3.2, a random threshold is chosen between the values of *L*1 and *L*2, after applying the EM algorithm. The chosen threshold is the result of the study shown in Figure 17. Here, a simulation of 10,000 randomly generated frames was performed for each SNR value. In this simulation, the two levels *L*1 and *L*2 were chosen as the maximum or the minimum threshold value, respectively, necessary to the NN training. In Figure 17, the average and STD of the frequency edges detection for each of the limits of this threshold, is shown. Here, choosing any of these limits does not represent a big change in the estimation of frequency edges, which means that if any random value is chosen between one of these two limits, it will not represent a big difference. In practice, it is extremely complicated to determine the optimum value for this threshold, because the premise of multiband spectrum sensing technique is based on the ignorance of the characteristics of the received signal (frequency edges, SNR, presence of UP, bandwidth, etc.) For SNR < −2 dB the proposed methodologies do not have a good performance since the signals are embedded in noise.

**Figure 18:**
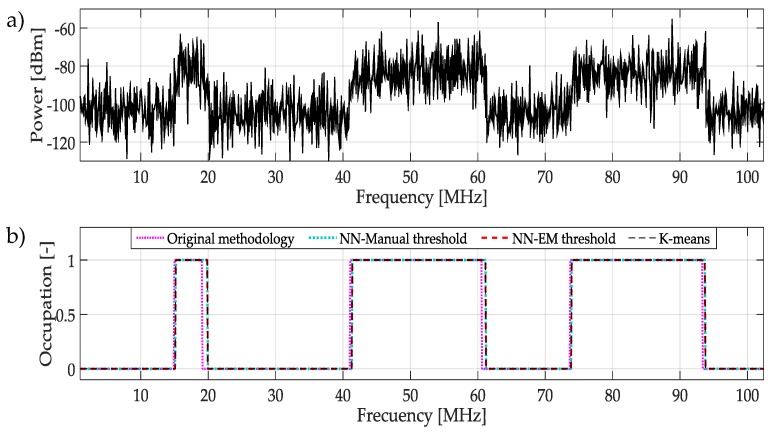
(**a**) Multiband spectrum generated randomly. (**b**) Original methodology and proposed methodologies applied to determine the occupation by PUs. Where all of them detect correctly UPs transmissions.

In Figure 18a, it is highlighted a random multiband spectrum that represents a challenging case because of the contained power noise. In Figure 18b, we can observe that the three methodologies have an excellent performance in a quite noisy environment. 

The frequency edges for the original methodology and the different proposals for the example of Figure 18, are shown in Table 2. The edges where the transmissions of the PUs were placed in the simulation have been also included. It is clearly observed that the proposals presented in this paper prove to be more accurate than the original methodology.

**Figure 19:**
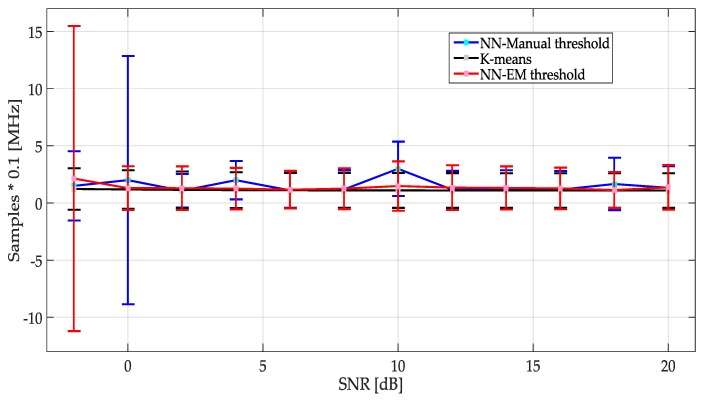
Mean and STD for each method. It is noteworthy that *K*-means shows a constant behavior, compared with the others.

Figure 19 shows the mean and STD of the frequency edges detection as a function of the SNR for each proposed ML technique described in Section 3. Apparently, the mean of each of them tends to have the same behavior; however, the variance for each SNR differs in some of the cases. Table 3 shows the values of mean and STD, where it is possible to appreciate how for higher SNR values all ML techniques tend to similar values, being *K*-means the most stable method. 

**Figure 20:**
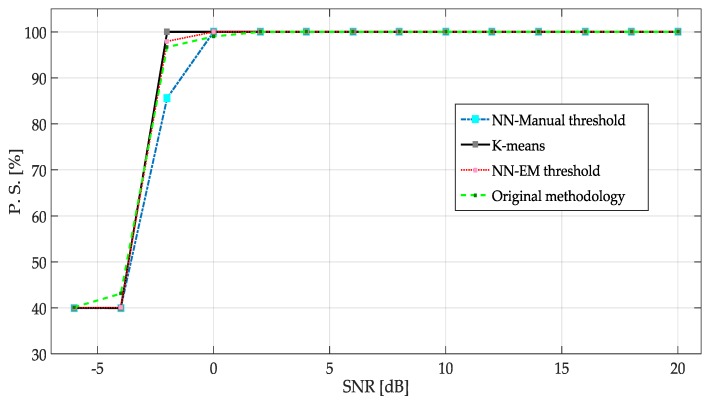
PS of each of the proposed methodologies.

The percentage of success (PS) for each methodology is plotted in Figure 20. Some important points can be remarked:

- The performance of NN with manual threshold, NN with EM threshold and *K*-means for SNR >= 0 have quite acceptable results, obtaining success rate practically around 100% of the correct detection.
- An improvement was observed for the NN that has been trained with EM just in SNR = −2 dB.

The proposals presented in this paper show a better performance than the original methodology (see Figure 20). Not only in PS, also, in the precision to detect the frequency edges (see Table 3).

### 4.2. Signals Obtained from the Environment 

**Figure 21:**
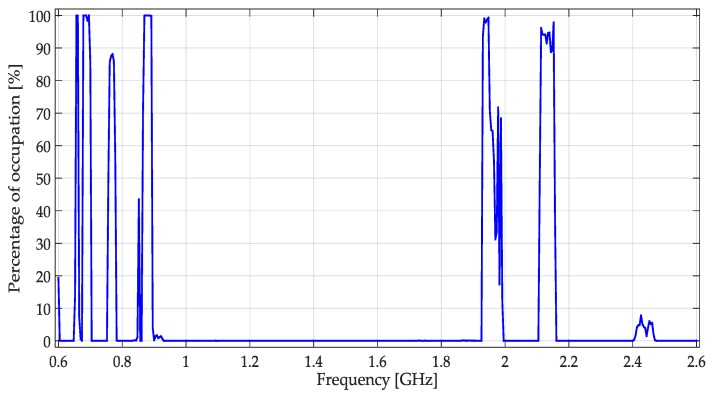
Percentage of occupation for a week in the frequency interval [0.6–2.6] GHz presented in [10].

The real signals mentioned in Section 4.3 of [10] were again used to test the performance of different proposed methodologies. These signals were obtained from a whole band varying from 0.6 GHz to 2.6 GHz. The percentage of occupation in this wideband is highlighted in Figure 21. Table 4 presents the specific bands measured and for which services are they assigned.

### 4.3. Results from the Real Signals

**Figure 22:**
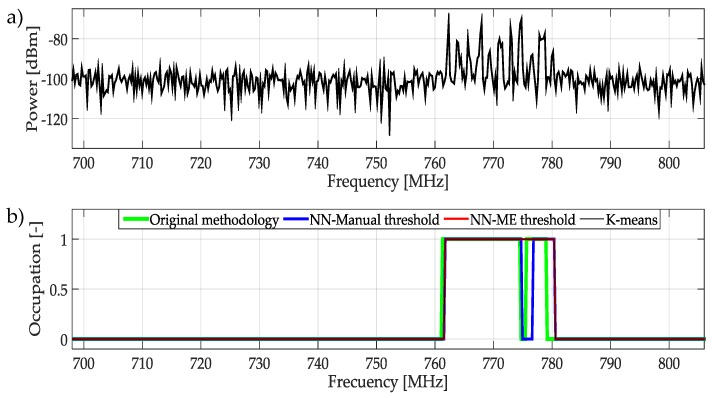
(**a**) A real signal obtained from the frequency band [698–806] MHz. (**b**) The result of applying the four proposed methodologies to the real signal in terms of spectrum occupancy.

**Figure 23:**
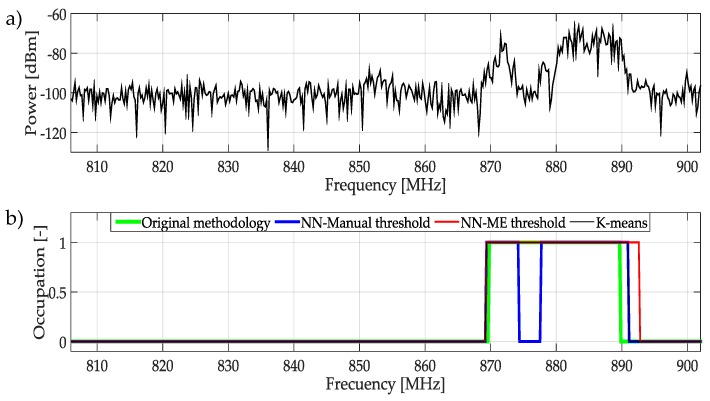
(**a**) A real signal obtained from the frequency band [806–902] MHz. (**b**) The result of applying the four methodologies to the real signal in terms of spectrum occupancy.

**Figure 24:**
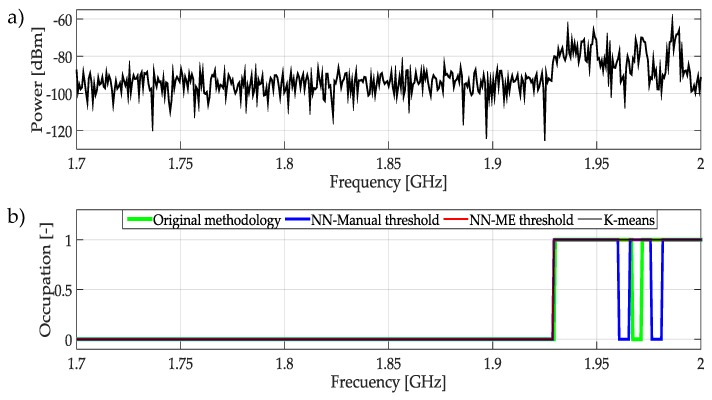
(**a**) A real signal obtained from the frequency band [1.7–2] GHz. (**b**) The result of applying the four methodologies to the real signal in terms of spectrum occupancy.

**Figure 25:**
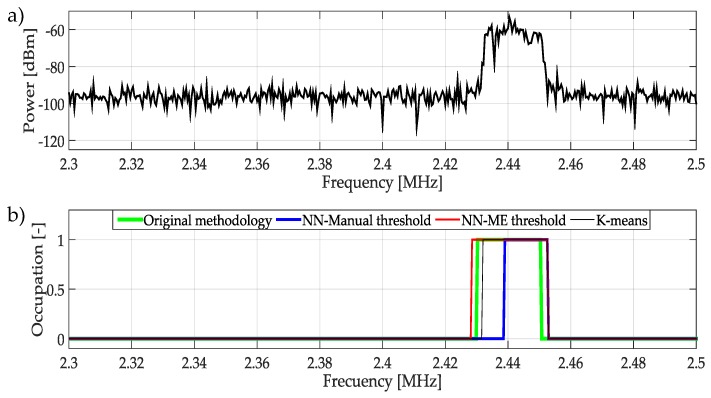
(**a**) A real signal obtained from the frequency band [2.3–2.5] GHz. (**b**) The result of applying the four methodologies to the real signal in terms of spectrum occupancy.

**Figure 26:**
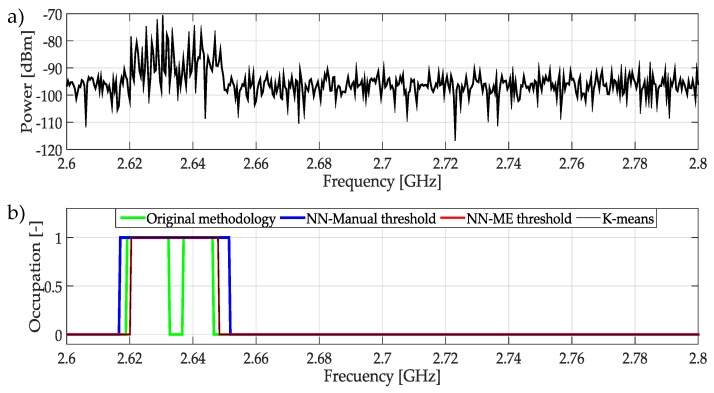
(**a**) A real signal obtained from the frequency band [2.6–2.8] GHz. (**b**) The result of applying the four methodologies to the real signal in terms of spectrum occupancy.

The real signals obtained from the environment are totally unknown, being this kind of scenarios a challenge regarding the right estimation of the set of frequency edges and power transmission of PUs. Nevertheless, after some signal processing, it is possible to infer the presence of PUs. The methodology described early in this paper and in the previous work presented in [10], it is examined with the ML techniques considering the different bands of Table 4. In the next, it is shown a challenging signal for each band. The results of PU detection are plotted in Figure 22, Figure 23, Figure 24, Figure 25 and Figure 26. 

The frequencies examined from 698 MHz to 806 MHz appear in Figure 22a. This signal presents a non-constant power transmission of PUs around 760–780 MHz. In practical terms, this kind of activity determines totally occupied this bandwidth. Thus, ML techniques and the original methodology detect frequency space as occupied as can be seen in Figure 22b.

The signals observed in the frequencies of 806 MHz to 910 MHz are shown in Figure 23. In this figure, most of the transmission signals are detected by all techniques. NN with manual threshold provides the best results. This technique detects a signal with low power that apparently corresponds to a PU. In contrary case, the original methodology does not detect changes on power and the bandwidth size detected is lower.

The frequencies measured from 1.7 GHz to 2 GHz are presented in Figure 24a. Here, in general, the occupied and empty bands are detected correctly by the ML techniques as can be seen in Figure 24b. However, in the range from 1.925 GHz to 2 GHz there are several transmissions. The NN with manual threshold techniques detects these changes in the occupation of the band. The frequency edges are not detected with high precision, but this technique is more sensitive to these changes.

Figure 25a shows the frequencies observed between 2.3 GHz and 2.5 GHz. In this case, the three techniques provide affordable results classifying the occupation of the signal as can be seen in Figure 25b. In this signal, all techniques show good performance in front of PU transmission.

In the case of the frequency range from 2.6 GHz to 2.8 GHz shown in Figure 26, all techniques provide good results detecting PUs. However, being *K*-means the one that shows the best detection of frequency edges.

## 5. Conclusions

In this work, the frequency edges detection phase, mentioned in the methodology proposed in [10], was improved based on ML techniques. First, a NN with a manual threshold in the training phase was implemented, allowing a more accurate classification of the approximation coefficients. Similarly, applying EM to NN contributes to increase the effectiveness of the training phase. Besides this technique avoids the intervention of another user to set a manual threshold of the NN training. That is, we passed from a supervised technique to a fully automated algorithm. 

From the results of the phase of detection of frequency edges and PS to detect a PU presence correctly, we can conclude that *K*-means is the best ML technique analyzed in this work. It is also important to remark that an improvement in the detection, with signals in a noisy environment, is observed. Additionally, this method does not require a great computational complexity, comparing with an NN that requires a more advanced hardware power.

Throughout the different simulations, it has been perceived that the precision does not depend only on the used ML technique nor on the chosen method of spectrum sensing, it also depends on the number of samples of the signal to be evaluated. Indeed, given that processed elements are not directly the samples of the spectrum signal X(f) but its approximation coefficients (obtained from the multiresolution analysis), that means the number of points of the signal X(f) is reduced by $2^{L}$ where L is the level of decomposition in the MRA. Hence, it is noteworthy that the proposed methodologies were tested on signals recovered from the work environment that only has 461 samples, which means that only 57 approximation coefficients will be obtained. Even though this number of samples is quite small for a multiband environment, the results of the methodology implemented in this work are quite good. As part of future work, the complete methodology considering the ML techniques are thought to be implemented in a software defined radio platform. Also, to get an accuracy description of proposed algorithms where parameters as evacuation time can be measured, a cooperative cognitive radio network is going to be developed [27].

## Figures and Tables

**Table 1:** Simulation parameters.

	ML Technique		
	NN-Manual Threshold	NN-EM Threshold	*K*-Means
Software	MATLAB 2014b		
Value for SNR	−6 a 20 dB spaced by 2 dB		
Number of frames per each SNR value	500,000		
Number of symbols per each frame	Randomly between [0, 2]		
Bandwidth	102.4 MHz		
Samples per frame	1024		

**Table 2:** Frequency edges detection with the different techniques of the example shown in Figure 18.

	Frequency Edges Detected [MHz]							
Simulation	0.1	14.2	19.4	40.7	60.7	73.7	93.7	102.4
Original methodology	0.1	14.2	18.4	40.5	60.2	73.4	93.2	102.4
NN-Manual threshold	0.1	14.4	19.2	40.8	60.8	73.6	93.6	102.4
NN-EM threshold	0.1	14.4	19.2	40.8	60.8	73.6	93.6	102.4
*K*-means	0.1	14.4	19.2	40.8	60.8	73.6	93.6	102.4

**Table 3:** Mean and STD of frequency edges detection per SNR.

Technique		SNR											
		−2	0	2	4	6	8	10	12	14	16	18	20
NN-Manual threshold	Mean	1.49	2.00	1.07	1.99	1.10	1.18	2.99	1.16	1.18	1.16	1.66	1.33
	STD	3.03	10.86	1.47	1.68	1.53	1.67	2.38	1.64	1.67	1.64	2.29	1.90
*K*-means	Mean	1.22	1.18	1.15	1.12	1.11	1.10	1.10	1.10	1.09	1.09	1.09	1.09
	STD	1.81	1.68	1.61	1.57	1.55	1.53	1.52	1.52	1.51	1.51	1.51	1.51
NN-EM threshold	Mean	2.14	1.30	1.29	1.26	1.17	1.25	1.48	1.35	1.32	1.27	1.13	1.36
	STD	13.34	1.92	1.91	1.81	1.64	1.79	2.15	1.95	1.89	1.82	1.58	1.96

**Table 4:** Monitored frequency bands presented in [10].

Frequency Band	Type of Communication
[698–806] MHz	Mobile and landline.
[806–902] MHz	Mobile and aeronautical mobile.
[1.7–2] GHz	Mobile and fixed.
[2.3–2.5] GHz	Radiolocation, amateur, mobile and fixed.
[2.6–2.8] GHz	Aerial vehicles, radiolocation and, radio navigation.

## Abbreviations

CR	Cognitive radio
SDR	Software-defined radio
DSA	Dynamic spectrum access
SU	Secondary users
PU	Primary users
SS	Spectrum sensing
MSS	Multiband spectrum sensing
WBSS	Wide-band spectrum sensing
MRA	Multiresolution analysis
HFD	Higuchi fractal dimension
ML	Machine learning
RL	Reinforcement learning
NN	Artificial neural networks
SVM	Support vector machine
CSS	Cooperative spectrum sensing
EM	Expectation maximization
NAC	Normalized and interpolated approximated coefficients
TW	Test window
STD	Standard deviation
PS	Percentage of success
